# Impact of drought on mental and behavioral disorders, contributions of research in a climate change context. A narrative review

**DOI:** 10.1007/s00484-024-02657-x

**Published:** 2024-03-19

**Authors:** Alicia Padrón-Monedero, Cristina Linares, Julio Díaz, Isabel Noguer-Zambrano

**Affiliations:** 1 Health Programs Department, National School of Public Health, Carlos III Health Institute, Av./ Monforte de Lemos 5, 28029 Madrid, Spain; 2 Climate Change, Health and Urban Environment Reference Unit, National School of Public Health, Carlos III Health Institute (Instituto de Salud Carlos III/ISCIII), Av./ Monforte de Lemos 5, 28029 Madrid, Spain

**Keywords:** Droughts, Suicide mortality, Mental disorders, Environmental factors, Heat waves, Climate change

## Abstract

Mental and behavioral disorders are an important public health problem and constitute a priority for the WHO, whose recommendations include the surveillance of their risk factors. On the other hand, drought episodes have been increasing in frequency and severity in Europe since 1980. Therefore, to review the present knowledge about the impact of drought on mental and behavioral disorders, in the present climate change context, and to underline potential research gaps, could be of major interest. Thus, we performed a narrative review using online academic databases with the aim of identifying relevant literature about the impact of drought on mental and behavioral disorders. To the best of our knowledge, no study in Europe quantifies the potential association between drought and mental disorders. A limited number of studies have found significant associations between droughts (with different temporal ranges) and various measures of mental health. However, according to our review, only three of them quantified the association between drought and objective mental health outcomes, such as number of emergencies due to clinically diagnosed mental disorders or suicides. Additionally, few studies used specific indices as a measure of drought; and finally, as far as authors are aware, none of them has analyzed this relationship adjusting for various other potential environmental confounders. Moreover, the eventual association could vary between different geographical areas within the same country. Therefore, national and regional studies would be especially necessary. Thus, there is a need for specific national and regional studies, in Europe and globally, that assess the impact of specific indices of drought (with different temporal ranges) on objective mental health outcomes controlling for potential environmental confounders. Moreover, the quantification of its cost would be necessary for health prioritization, evidence-based policies and strategic health planning.

## Introduction

Mental and behavioral disorders are an important public health problem and constitute a priority for the WHO, whose recommendations include that mental health should be included in the research agenda on climate change (World Health Organization 2022a), and also its risk factors surveillance (World Health Organization 2013). However, despite drought episodes have been increasing in frequency and severity in Europe since 1980, and that according to the Spanish Meteorological Agency (AEMET) reports, the hydrological year 2021–2022 was the third driest of the current century and the sixth of the entire historical series (AEMET 2022); only a limited number of studies have quantified the association between droughts and various measures of mental health, finding significant associations (Yap et al. 2021; Hanigan et al. 2012; Nicholls et al. 2006; Luong et al. 2021; Obrien et al. 2014; Hanigan et al. 2018; Austin et al. 2018). Since variations have been described for different geographical areas (Yap et al. 2021; Hanigan et al. 2012; Obrien et al. 2014), for different measurements (of both drought and mental health) (Luong et al. 2021; Obrien et al. 2014; Hanigan et al. 2018; Austin et al. 2018; Powers et al. 2015) and some environmental variables could be potential confounders of the association between drought and mental disorders (Hanigan et al. 2012); we aim to review the present knowledge about the impact of drought on mental and behavioral disorders, in the present climate change context, and to underline potential research gaps.

### Mental health burden

Mental and behavioral disorders have a remarkable prevalence with estimates of around 970,812,400 individuals worldwide (James et al. 2018), and they will involve economic losses of 16.3 billion dollars between 2011 and 2031 (Bloom et al*.* and World Economic Forum 2011). In Spain, in 2017, around, 19% of the population presented psychological distress (assessed by the General Health Questionnaire-12), 15% received some type of diagnosis of mental disorder (Henares Montiel et al. 2020), and the adjusted suicide mortality rates (2011–2015) were 8.2 per 100,000 population (de Pedro Cuesta et al. 2017). Additionally, for several European countries, including Spain, approximately 25% of the population will suffer from a mental disorder at some point in their life (Alonso et al. 2004). Finally, WHO urges member states to "develop surveillance frameworks that include risk factors and social determinants of health to analyze and assess trends with respect mental disorders" (World Health Organization 2013).

### Climate change and mental health

The United Nations Framework Convention on Climate Change (UNFCCC) states that Climate Change “means a change of climate which is attributed directly or indirectly to human activity that alters the composition of the global atmosphere and which is in addition to natural climate variability observed over comparable time periods” (United Nations 1992; Intergovernmental Panel on Climate Change (IPCC) 2014). And the Working Group II of the Intergovernmental Panel on Climate Change (IPCC) assesses as important impacts of Climate Change, increases in the frequency of extreme temperatures (heat waves) and extreme events like droughts and floods, among others (Romanello et al. 2021; Intergovernmental Panel on Climate Change 2014). More specifically, they state that by the end of the twenty-first century there would be a global and regional increase in intensity and duration of droughts (Intergovernmental Panel on Climate Change 2013). Specifically, it has been described that by 2050 global warming is likely to increase global temperature by 1.5 or 2ºC (Cammalleri C et al*.* 2020) and in specific sub-regions, like west-central Europe, an increase of temperature even under 2 ºC would increase drought severity between the 20% to 39% (Aalbers et al. 2023).

The specific impact of climate change in mental health includes: a worsening of psychological distress, (Palinkas and Wong 2020; Charlson et al. 2021; Lawrance et al. 2021, 2022; World Health Organization 2022b), sleep disturbances, (Lawrance et al. 2021), lethargy and cognitive impairment (Palinkas and Wong 2020), anxiety (World Health Organization 2022b), solastalgia, (World Health Organization 2022b; Lawrance et al. 2021) increases in mental disorders incidence (World Health Organization 2022b; Obradovich et al. 2018; Charlson et al. 2021; Lawrance et al. 2021, 2022) and exacerbation of symptoms, (World Health Organization 2022b; Lawrance et al. 2021), greater susceptibility to diseases and mortality for those with previous mental illness (Charlson et al. 2021; Lawrance et al. 2021) and increased suicide mortality (World Health Organization 2022b; Palinkas and Wong 2020; Charlson et al. 2021; Lawrance et al. 2021, 2022). Additionally, climate change is related to events that may have long-term consequences (degradation of ecosystems and even loss of the environment, (World Health Organization 2022b), economic and social alterations, migration and increased conflicts (World Health Organization 2022b)) that may be associated with mental problems (Lawrance et al. 2021; United Nations High Commissioner for Human Rights 2016). For example, the projections have estimated that globally for 2050, there will be roughly 200 million climate refugees (Myers 2002), a situation that may have consequences in undermining their social support and their mental well-being. (Trombley et al. 2017; Adger et al. 2013).

Thus, accurate meteorological forecasts related to climate change (including droughts) and their specifics impacts in mental health are necessary in order to consider them in the development and implementation of efficient national health and climate change plans to mitigate its expected burden (Romanello et al. 2021).

### Drought impact on human health other than mental health

The detrimental impact of droughts on human health, other than mental health, has been evidenced by several studies that have found significant associations with cardiovascular problems (Berman et al. 2017), respiratory problems (Yusa et al. 2015), cancer (Stanke et al. 2013) increased risk of certain infectious diseases (Yusa et al. 2015; Stanke et al. 2013; Salvador et al. 2020a) and mortality from different causes (Berman et al. 2017; Salvador et al. 2020a; Salvador et al. 2020b; Salvador et al. 2020c; Salvador et al. 2021). Moreover, extreme temperatures, that are related to droughts, have additionally been associated to worsening of cerebral and gastrointestinal diseases, to diabetes and to renal disorders (Marí-Dell'Olmo et al. 2022).

## Methods

The review question addressed by our group was: What is the current evidence available about the impact of drought on mental and behavioral disorders? Consequently, the objective of this review was to identify relevant literature, across the disciplines of drought and mental health, and summarize it in a comprehensive narrative review with the aim of providing the current evidence about the impact of drought on mental and behavioral disorders, and to underline potential research gaps.

As a search strategy (including the criteria for inclusion and exclusion), we performed two searches in PubMed, EMBASE ((Excerpta Medica Data Base) and Cochrane Collection (initial search in February 02 2022 and an updated search in February 2023 in order to include the last papers published during the drafting of the manuscript) for peer-reviewed qualitative or quantitative original articles and reviews in English or Spanish. The PubMed search string was Search ( (drought[Title/Abstract])) AND ((mental health[Title/Abstract]) OR (mental disorder[Title/Abstract]) OR (behavioral disorder[Title/Abstract]) OR (mental illness[Title/Abstract]) OR (suicide[Title/Abstract]) OR (self-harm[Title/Abstract]) OR (psychological distress[Title/Abstract])).

No further restrictions were included in the search. Additional studies (including peer-reviewed articles, books, and documents from official international organizations) were identified from reference lists of key relevant articles. We also reviewed documents from official international organizations suggested by co-authors and collaborators from our networks. Additionally the information about “3. Mental health burden” “4. Climate Change and mental health” and “5. Drought impact on human health other than mental health” was obtain from former bibliographic searches from our study group that were included in previous publications (Gómez González et al. 2023; Linares Gil et al. 2022).

Duplicate articles were removed prior to the selection process. The identified articles were first subjected to a title/abstract screening for eligibility (explicitly studying mental health and drought variables, adequate metrics to capture drought, adequate metrics to capture mental health, focused on the explicit link between drought and mental health, methodological quality, provided relevant information…) and then to a full text screening by APM. No specific validated tool was used to assess the quality of the selected studies. In specific cases, determinations for its final inclusion were made after consulting with the other coauthors.

Data from selected articles were extracted into a descriptive table with the following information: Reference; country; population; exposure variable; outcome variable; study design; main results.

## Drought impact on mental health

### Drought impact on mental health. European research

Although drought episodes are increasing in frequency and severity in Europe since 1980 (European Commission 2010), and according to the AEMET reports, the hydrological year 2021–2022 was the third driest of current century and the sixth of the entire historical series (AEMET 2022); to the best of our knowledge there are no national studies, neither in Spain nor in Europe, that quantify the potential association between drought and mental and behavioral disorders.

### Drought impact on mental health according to different mental health measurements and drought indices

According to our review, the only specific study that quantitatively analyzed the relationship between droughts and emergencies due to mental problems (Yap et al. 2021), was conducted in Australia and found significant associations for the winter periods. Also in relation to mental health problems two other studies, also in Australia, found significant associations between droughts and suicide mortality (Hanigan et al. 2012; Nicholls et al. 2006). It is interesting to highlight that the studies of Yap et al*.* (Yap et al. 2021) and Nichols et al*.* (Nicholls et al. 2006) used the amount of precipitation as a measure of drought, instead of specific indices.

A limited number of studies, all of them in Australia, and mainly in rural population, have additionally analyzed the association between drought and psychological distress (Luong et al. 2021; Obrien et al. 2014; Hanigan et al. 2018; Austin et al. 2018). The ones that used specific indices to assess drought, found significant associations for the global population or different subpopulations (Luong et al. 2021; Obrien et al. 2014; Hanigan et al. 2018). Nevertheless, Austin et al*.* (Austin et al. 2018) did not find associations between rainfall and psychological stress, although they did find associations with community stress related to drought. Finally Powers et al*.* did not find significant associations between droughts, assessed with the Hutchinson Drought Index, and mental health assessed by the Mental Health Index in rural Australian women (Powers et al. 2015).

### Potential environmental confounders

No study, according to our review, has analyzed the relationship adjusting for other environmental variables (with the exception of temperature in a specific study (Hanigan et al. 2012)) that could be potential confounders of the association between drought and mental and behavioral disorders. For example, heat waves (Obradovich et al. 2018; Díaz et al. 2020; Lawrance et al. 2021), cold waves (Díaz et al. 2020), air pollution (Lim et al. 2012; Szyszkowicz 2007; Lawrance et al. 2021) and noise pollution (Díaz et al. 2020) have been associated with worse mental health; and additionally, practically all of these environmental phenomena, are frequently linked to episodes of drought.

### Temporal relationship between drought and mental health

Another important factor to consider is the temporal relationship between drought and mental health. The scarce bibliography that has analyzed this relationship in Australia, has assessed a potential impact of drought on mental health in the short, in the medium and in the long terms (Yap et al. 2021; Hanigan et al. 2012; Nicholls et al. 2006; Luong et al. 2021; Obrien et al. 2014; Hanigan et al. 2018). Additionally, it has been suggested that the duration of drought could determine the degree of mental disturbance (from psychological discomfort to severe mental disorders) (Yap et al. 2021). But, on the other hand, Luong et al. assessed that for the first 2.5–3 years of drought, psychological distress increases, but after the third year gradually starts to decrease, following an inverted U-shape (Luong et al. 2021).

### Geographical variations

Lastly, the potential association between drought and mental health disorders could vary between different countries (with different geographical, environmental, socioeconomic and health characteristics), given the variations that have been described for different geographical and socioeconomic areas within the same country (Yap et al. 2021; Hanigan et al. 2012; Obrien et al. 2014). Thus, specific national and regional studies are needed to analyze the relationship in different geographical areas.

### Biological plausibility

Drought episodes can affect mental health mainly through two biological plausibility mechanisms:

#### Pathophysiological

Drought is considered a psychological stressor (Yusa et al. 2015; US climate resilience toolkit 2019) and even just the threat of climate change and its expected consequences, including drought, are important psychological and emotional stressors (Marí-Dell'Olmo et al. 2022; Dodgen et al. 2016), especially for the younger population, that report, among others, feelings of sadness and anxiousness that affect their daily life (Hickman et al. 2021). Moreover, important hormonal and metabolic pathways related to chronic psychological stress can affect human health (Epel 2009). Specifically, there have been found associations between chronic psychological stress, hypothalamic–pituitary–adrenal axis dysfunction, and depression (Epel 2009). Additionally, psychological stressors are associated with chronic inflammation (Epel 2009; Singh and Newman 2011) and oxidative stress (Epel 2009; Schiavone et al. 2015), which in turn are considered pathophysiological mechanisms that contribute to the development of mental and behavioral disorders (Kivimäki et al. 2014; Schiavone et al. 2015) as well as numerous non-communicable diseases (Seyedsadjadi and Grant 2020). However, no study has specifically analyzed the association between droughts and an increase in inflammatory markers, oxidative stress, telomere shortening and/or alteration of the telomere-mitochondrial cell aging axis; in the way it has been assessed for other environmental factors closely linked to drought episodes (such as environmental pollutants (including dust particles) (Martens and Nawrot 2016; Elbarbary et al. 2021; Niemann et al. 2017), the fires (Koopmans et al. 2022) and heat waves (Gostimirovic et al. 2020)). Specifically, it has been shown that heat waves can produce systemic inflammation, increase of free radicals and increase of neuronal apoptosis; and they can also modify the pharmacokinetics of certain drugs, which has a particularly serious impact on psychiatric patients (Gostimirovic et al. 2020). Likewise, fires have also been associated with increased systemic inflammation and oxidative stress (Koopmans et al. 2022). Finally, drought stresses water availability, reduces water quality with increase in contaminant and pathogen concentrations (Yusa et al. 2015) and is related to the scarcity of food leading to food/water insecurity and malnutrition (Yusa et al. 2015; Vins et al. 2015; Cianconi et al. 2020; Alpino et al. 2016). These factors may impact physical health by: leading to gastrointestinal illness, transmittable diseases, iron and other nutritional deficiencies (Yusa et al. 2015; Alpino et al. 2016) that, among other consequences, can weaken the immune system (Alpino et al. 2016), and also to other physical disorders (Alpino et al. 2016). Those physical disorders may have a reciprocal relation with mental health (Yusa et al. 2015; Berry et al. 2010). Moreover, drought is also related to hydroelectricity power shortages that may alter health protective temperatures (Yusa et al. 2015) that may be related with a worse mental health (Obradovich et al. 2018; Díaz et al. 2020; Lawrance et al. 2021).

#### Psychological

Numerous interrelated and complex causal pathways have been described between drought and mental disorders (Vins et al. 2015; Berry et al. 2010) that include aspects of economic loss, uncertainty about the future or about the accessibility of basic resources, social isolation, deterioration of community well-being, work overload, family stress, solastalgia (sadness due to the degradation of the environment, home and feeling of belonging) and migration to areas with better employment opportunities with the subsequent breakdown in social support, acculturation and resistance by receiving communities among other difficulties (Vins et al. 2015; Berry et al. 2010; Yusa et al. 2015; Alpino et al. 2016; Cianconi et al. 2020).

Specifically, among the societal changes due to drought, it has been described that economic loses and unemployment due to drought are related to political instability (Gleick 2014). Furthermore, there have been described numerous water-related conflicts (Gleick 2014). Additionally, the economic difficulties related to drought may produce changes in behavior (like social isolation (because of humiliation and shame), increasing workloads and subsequent decreased time to interact with family and friends, and increasing the tension within the family that could lead to domestic abuse) (Vins et al. 2015). Finally, would be of interest to assess whether mental health could be both an important underlying mediator and an outcome for some of those behavioral and sociopolitical disorders.

### Economic quantification of the impact of drought on mental health

As far as we know, no economic study has assessed the cost of drought in terms of health impacts, including mental health (Schmitt et al. 2016). Although, It has been described that for the EU plus the UK the estimated general annual losses due to drought for the period 1981–2010 were 9.0 €billion/year (CI 95%: 7.4–14.2 €billion/year) and for 2100 the projected economical loses due to drought would be 24.7 (20–35) €billion/year for only 1.5 °C increase in global temperature (Cammalleri et al. 2020). Additionally, in policy-making the impact of climate change (including drought) on mental health and its health cost burden are underestimated (Lawrance et al. 2022), for this reason the potential benefits of policies and/or actions that limit climate change (including drought) would be greater than expected (Lawrance et al. 2022; Linares Gil et al. 2022). Thus, in addition to the impact of the drought on mental health, the economic quantification of the cost of drought in terms of mental health impacts could be especially necessary to guide health actions (in terms of prioritization, organization, care resources and the ability to anticipate risks, among others), as well as public health policies (including suitability and prioritization of potential preventive actions) and environmental policies, with the objective of reducing its burden in economic and health terms. Moreover, this knowledge will be needed for the 2022 International Drought Resilience Alliance that has the support of United Nations (United Nations 2022).

## Limitations

This narrative review has several limitations:

### Limitations of the reviewed literature

First, some of the articles included in the review, used as an outcome variable measures of mental health that were self-reported, consequently they could include a potential declaration bias. Although, since the number of articles reporting objective measurements of mental health (suicide, hospital admissions for mental health problems…) was low, we decided also to include and describe in our study the information from these papers. Second, some articles did not use specific indices to assess drought. Third, some articles included in the review analyzed data from specific populations (for example rural women). Although, all the information about the measurement of the exposure, the outcome variables and the population analyzed was acknowledge and described in the review. Fourth, as far as we know only one study (Hanigan et al. 2012) analyzed the relationship adjusting for other environmental variable that could be potential confounder of the association between drought and mental health. Fifth, most of the selected studies were ecological and, therefore, causality could not be inferred at the individual level.

### Limitations of the narrative review

First, the quality assessment for the selection of articles relied mostly in the research expertise of the authors, that although they have a large research experience both in mental health and in the environmental fields, this quality assessment was not performed with a specific validated tool, thus it could be considered partially subjective. On the other hand, most of the studies had unique study designs and Charlson et al. (Charlson et al. 2021) reported that the existing quality assessment tools, for scoping reviews about climate change and mental health, were not exactly tailored to the type of designs used in these kind of research papers. This conclusion could be extrapolated to the difficulty to perform a perfectly objective quality assessment in our review. Second, since our search only included literature in English or Spanish we cannot rule out that important papers not written in those languages could have been missed. Third, we did not include in the search criteria, specific mental and behavioral outcomes other than suicide, self-harm and psychological distress. Although given the small number of articles obtained from these, relatively easy to collect, outcomes it is unlikely that an important amount of relevant literature about other specific metal health domains (anxiety, depression, bipolar, suicide, schizophrenia) would have been missed. Fourth, almost all the studies were conducted in Australia, and some of them from specific geographic locations within the country, thus their results may not be extrapolated to other countries and/or regions. On the other hand, this limitation highlights the need for additional studies in other geopolitical locations.

## Conclusions

Limited number of studies, all of them in Australian populations, have assessed the impact of specific indices of drought (with different accumulation ranges) on objective mental health outcomes. As far as we know, only one study assessed the impact of drought on emergencies due to mental problems and two of them on suicides, finding all of them positive impacts. However, none of them adjusted for potential environmental confounders. Additionally, the potential association between drought and mental health disorders could vary between different countries (with different geographical, environmental, socioeconomic and health characteristics). Thus, there is a need for specific national and regional studies, in Europe and globally, that assess the impact of specific indices of drought on mental health. Moreover, the quantification of its cost is necessary for health prioritization, health policies and planning.

## Data Availability

Data sharing not applicable to this article as no datasets were generated or analysed during the current study.

## Abbreviations

WHO: World Health Organization
AEMET: Spanish Meteorological Agency
SPI: Standardised Precipitation Index
SPEI: Standardised Precipitation-Evapotranspiration Index
